# Recycling Waste Agricultural Nets as Cement Composites

**DOI:** 10.3390/ma17081828

**Published:** 2024-04-16

**Authors:** Bartosz Zegardło, Chrysanthos Maraveas, Kamil Świeczka, Antoni Bombik

**Affiliations:** 1 Faculty of Agricultural Sciences, University of Siedlce, B. Prusa 14, 08-110 Siedlce, Poland; ks82737@stud.uph.edu.pl (K.Ś.); antoni.bombik@uws.edu.pl (A.B.); 2 Department of Natural Resources and Agricultural Engineering, Agricultural University of Athens, Leof. Athinon 51, 104 47 Athens, Greece; c.maraveas@maraveas.gr

**Keywords:** agricultural nets, straw bale wraps, waste, recycling, filler, additive, cement composite

## Abstract

The advancement of agricultural mesh technology has contributed to its improved properties. As a result, agricultural nets are widely adopted in large-scale farming applications, for example, in cereal crop farming. However, a consequence of this increased use of agricultural nets is the accumulation of large amounts of waste. The current paper focuses on the recycling of agricultural nets used in wrapping straw bales to develop additives and fillers in cement composites. The research details an analysis of the use of waste agricultural meshes as an ingredient in cement composites. Six test series of different mixtures were conducted. In the first four series, agricultural waste was utilised as an additive in a composite comprising aggregate and cement slurry (the amounts of wasted nets were 20, 40, 60, and 80 kg/m^3^). In the last test series, the recyclate utilised comprised a mixture of cement slurry and waste only. The composites were subjected to standard tests and thermal resistance tests. The results showcased that that the addition of a net worsened the workability of the concrete mixture, and with increasing amounts of addition, the consistency of the mixture could change from liquid to dense plastic. The flexural strength of the composite decreased with increasing amounts of recyclate. In subsequent test series, the flexural strength value was lower than that of the control (3.93 MPa), from 7.38% (3.64 MPa) for the composite with 20 kg/m^3^ of recyclate to 37.66% (2.45 MPa) for the composite with of 80 kg/m^3^ recyclate. The flexural strength value of the net-filled composite without aggregate was very high (10.44 MPa), where the value obtained for the control composite was 62.36% lower. The results of the compressive strength test showed a decrease in this parameter with increasing amounts of additive. The value assessed for the control composite was 27.99 MPa. As expected, the composite that had no aggregate and consisted of only recycled filler had the lowest compressive strength. The value of this parameter was 13.07 MPa, and it was 53.31% lower than that of the control composite. The results of the tests of resistance to temperatures were similar to those recorded for the composites with polypropylene fibres. All composites demonstrated a significant decrease in their compressive and flexural strength after annealing. SEM imaging showed that the net fibres were closely bonded to the cement stone. Finally, it was concluded that recyclates performed best as fillers in lightweight composites with a low density, low absorption, high flexural strength, and satisfactory compressive strength.

## 1. Introduction

Since the Industrial Revolution, the development of agricultural mechanization has transformed crop and livestock production [1]. The use of engines and equipment replaced labour and helped to speed up work, hence increasing production efficiency. A consequence of this was the emergence of crop combinations focused on the production of specific products, in which work proceeded more efficiently, more quickly resulting in a product that was often of a higher quality, as well as cheaper in terms of the overall cost of its production [2]. The shift away from small farms and the production of small quantities of various products has meant that agriculture today presents itself as a strong industry. Agriculture requires not only typical agricultural knowledge, but also advanced engineering techniques in terms of construction, machinery equipment, and IT techniques for control and steering processes [3]. The implication is that the environmental impact of such farms has also changed [4]. The environmental impact of small, organic farms follows the natural cycle of matter [5]. Most of the waste is disposed of without human intervention and occurs naturally in unforced biodegradation processes. In modern times, it is being increasingly acknowledged that the large-scale crop and livestock production facilities of new versions of farms exert a negative impact on the environment, and this branch of the economy itself is considered as more destructive for the environment [6,7]. In addition to the increasing the energy intensity of production, which consumes natural energy resources and introduces pollutants and heat into the atmosphere, waste is also generated on farms. Artificially extending the growing season in colder climates and growing crops in areas that are unnatural for the plants and animal species concerned often require mechanisms to protect these crops from the cold using foil pavilions and artificial protective materials to protect plants from the sun [8]. The non-biodegradable protective materials used for these purposes, for example, are periodically deposited in landfills after use due to their non-existent recycling methods [9]. A similar case is observed with the packaging materials used in agriculture. The raw materials delivered to farms and the products generated require well-thought-out load-securing measures [10]. Due to the scale of production, packaging materials from agriculture are also emerging as challenging wastes.

Agricultural nets are products used in large quantities on farms and are often subject to rapid depletion. These nets serve many purposes in agriculture. Among these, we can distinguish between wind nets [11,12], which protect crops from the wind, and shade nets [13], which protect crops from the sun’s rays. On farms, nets are also used to protect crops from insects such as aphids [14], spiders [15], birds, and bats [16]. In some cases, agricultural nets have been claimed to positively impact the environment because of their ability to reduce pesticide usage. However, the problem of managing these products after use remains unresolved. A similar observation has been made with the use of nets for hail protection [17].

Another type of agricultural net is used to wrap bales, which are products used in balers [18]. This type of net is considered to be an innovative system for tying hay or straw bales. The use of these nets has eliminated the twine that was previously used. This net system has also increased the efficiency of the machines by reducing the time needed to tie the bales, hence generating a positive impact on the environment. Wrapping a bale with a net using the latest machines takes about six seconds, while with twine, it takes at least 90 s. The comparative fuel consumption of these machines is about 12% lower with nets. Furthermore, where favourable operating conditions are provided, a baling machine with a net can make about 20% more bales per hour. Bale netting [19] has many advantages: it makes it easier to store the baled material, reduces the absorbability of the material, as it forms a layer over which water flows, allows for better compaction of the material, and allows for the fitting of knives in the baler that break up the material into fragments of up to 3 cm.

Most of the nets used by farms to date are produced from non-biodegradable materials such as polypropylene [20], polyethylene (PE), polyvinyl chloride, and high-density polyethylene (HDPE) [21], which is also used for bale-wrapping nets. HDPE is a thermoplastic resin made from the chemical molecule ethylene. The resin is very lightweight and strong. When extruding the net from the resin, the molecules are laid along the net, as this strengthens the product when stretched. This property of the resin makes the net able to withstand the pressure of the material compressed in the bale. The production of agricultural nets begins with the extrusion of a wide plastic film. The film is then cut into narrower strips, which go onto looms where knitting takes place, resulting in the final net product. The described production process shows that the properties of these net fibres are similar to those of other film products made from HDPE.

Although technologies have already been developed to produce nets from biodegradable plastics [22], their current production is only around 1% [23]. Likely, this percentage will not increase significantly over time, since HDPE is claimed to be optimal at this point due to its ease of production and technical performance. HDPE is impermeable to water and other liquids and is not susceptible to UV radiation [24], making it more durable. HDPE has also been proven to have higher mechanical properties, such as a high modulus of elasticity, which are desired by users.

Diverse scholars have examined the use of agricultural nets, despite it being a novel research area [25,26,27,28,29,30,31]. Published studies have examined the mechanical properties of plastic nets and the types of polymeric materials used to manufacture these nets in agriculture [26], and also evaluated the effectiveness of different materials comparatively [27]. Research work being undertaken also concerns the development of new techniques for net manufacturing [28], the mapping of the circulation of agricultural waste produced from the nets [29], the optical properties for optimal shading [30], and the development of materials for recycling and their impact on agricultural sustainability [31]. More farmers are using agricultural nets due to the advancement of agricultural mesh technology and the enhancement of their properties. However, a consequence of their high usage is the increased waste generation from their use. In Poland, where the research was conducted, significant waste from agricultural nets is reported. The production of cereal crops leads to harvesting large quantities of straw. As a result, agricultural nets are used by farmers based on their speed and durability advantages during storage. A consequence is that increased piles of waste are reported across major farms.

Taking this into account, this paper analyses the recyclability of bale-wrapping netting. The research aims to propose a recycling system that will not require sophisticated technical measures such as melting and can be carried out locally. Therefore, this paper is focused on the use of waste in other broad industrial sectors, such as the production of building materials. The construction industry is one of the few industries in which materials are produced on a large scale and, most importantly, in their production [32,33,34,35], they can also draw substrates from recycled resources. Such solutions are in line with EU policy trends. Increasingly, the need to produce new products using waste is being emphasized—even if these products are, at least, no worse than those produced by traditional methods [36]. There are several research papers [37,38,39,40] that have elaborated on the possibility of using recyclates as an ingredient in cementitious composites [41,42,43,44,45,46]. These studies not only focus on obtaining unique products, but also on waste disposal [47,48,49]. Subsequently, the impossibility of the spontaneous biodegradation of waste [50,51,52,53,54,55] attracts attention. On the other hand, there are also many works demonstrating that recycling additives positively impact cementitious composites. The technical parameters positively affected by the use of recyclates are abrasion resistance [56], ultra-high strength [57], high-temperature resistance [58], high chemical resistance [59], heat storage capacity [60], and resistance to sewage effluent environments [61]. Such articles [62,63,64] have demonstrated that, with the right composition of components, even recycled ones [65,66], the use of recyclates in composites can lead to positive economic and environmental effects. These environmental advantages include eliminating the need for detailed waste treatment or thermal treatment, which releases detergent washing and heat deposits into the environment. These processes are also cheaper for businesses, as they can be carried out locally in concrete production plants, and the adaptation of the waste does not require any special techniques apart from the grinding of the recyclate.

One of the aspects that motivated this research was the similarity of the fibres created from net waste to the polypropylene fibres commonly used in cement composites. The use of this type of additive is mainly analysed in terms of the issue of the performance of cementitious composites at high temperatures [67,68,69,70,71,72]. The use of fibres is proposed in this research in terms of composites exposed to rapidly rising temperatures and the associated phenomenon of the thermal spalling of concretes [73]. One of the explanations for this phenomenon [74] is the boiling of water vapours contained in the capillaries of concretes, which increase in volume at temperatures above 100 °C [75]. This phenomenon induces tensile stresses in the closed capillaries of concrete [76], and when the tensile strength of the capillary walls is exceeded, it causes the explosive stripping of concrete fragments [77]. This phenomenon affects airtight and compact concretes with low porosity, in which water vapours have no free migration paths [78,79]. The solution to this problem, as presented in research papers, is the use of fibres that dissolve at high temperatures and leave a reservoir of space for increasing the volumes of water vapour [80,81,82].

Many studies have focused on the use of plastic waste in cementitious composites [83,84,85,86] and the effects of these additives on the technical parameters of the concretes obtained. An analysis of consistency issues in fresh concrete mixes revealed that this type of additive had a negative impact on the workability of the concrete [87,88,89,90,91,92,93]. Skominas et al. [94], in their research work, proved that replacing 15% of fine aggregate with plastic resulted in a 40% lower workability. Due to the low volumetric weight of plastics, it was anticipated that the effect of this addition would lower the specific density of the composite. The authors of the research papers [95,96,97,98,99] unanimously found that the volumetric density of the composites decreased as the amount of recyclates added increased. In assessing the degree of density decrease, a reference can be made to the work of Ismail and Al-Hashmi [100], who, after testing samples containing 10%, 15%, and 20% plastic waste, found that the density of the composite decreased by 5%, 7%, and 8.7%, respectively. Based on the low absorbency of HDPE, it can be presumed that it will also influence the lower absorbency of the composite it is made with. Meena et al. [101], in their study, confirmed this assumption and also pointed out that a lower water absorption would reduce the permeability of the composite while increasing its durability [102,103]. Strength studies have shown that the addition of plastic recyclates generally leads to reductions in concrete compressive strength, tensile strength, modulus of elasticity, and unit weight [104,105]. Pereira et al. [106] demonstrated that the main influence on the compressive strength of a composite with recycled plastic is due to the volume that the recycled plastic occupies in the concrete mix. As the volume increases, the strength decreases. In terms of tensile and flexural strength testing, the influence of this characteristic depends mainly on the type of recyclate. Despite observed decreases in strength, some studies have stated that the addition of recyclates can positively impact flexural strength and increase with an increasing plastic content [107,108,109,110,111]. Interesting research results have also been presented in papers dealing with the effect of plastic fibres on the high-temperature resistance of composites. Mohammadhosseini and Yatim [112] revealed that the addition of plastic fibres had a positive effect on composites exposed to 800 °C. The addition was effective in reducing thermal spalling. Similar conclusions were drawn from the study by Girardi et al. [113]. Subsequently, composites containing 0.5% recycled fibres had no damage after exposure to temperatures of the order of 450 °C, while the same concrete without fibre additives cracked at temperatures of the order of 150 °C. In addition, in [114], the authors proved that composites containing plastics had a lower thermal conductivity, and in [115], the effect of the melting of synthetic fibres was confirmed.

The current research proposed the innovative utilization of waste agricultural nets as a filler and additive for cement composites. The novelty of this research stems from the fact that it is the first study that has addressed the use of waste agricultural nets for the composition of composites, and no previous study in the literature has focused on this area. The research proposal was based on the evaluation of past studies, where hypotheses regarding the justification of plastic fibres as cement composites were highlighted. A hypothetical positive impact of parameters on the flexural strength and heat resistance of the composites was highlighted.

## 2. Materials and Methods

### 2.1. Materials

**Figure 1:**
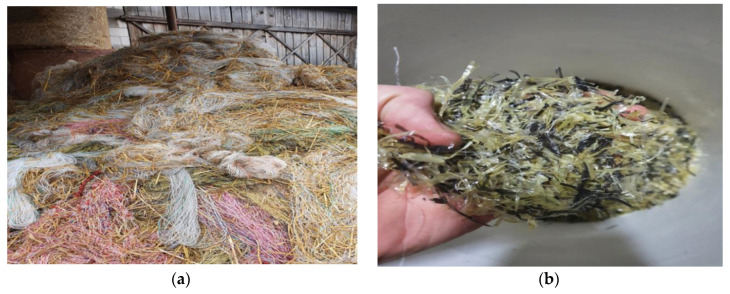
Waste netting for bale wrapping: (**a**) heaps of used netting at the farm and (**b**) netting in the form of fibres as dispersed reinforcement in the designed composites.

The main component targeted in the research work was waste agricultural netting used for wrapping straw or hay in specialized baling machinery. The waste was collected from a waste heap located in a functional farm (Figure 1a). The waste considered in the research was manually cleaned to remove straw impurities and, thereafter, sub-divided into individual 5 cm long fibres (Figure 1b).

Based on the available information, the technical parameters of this material (Table 1) proved that it could potentially have a positive effect on selected characteristics of the cementitious composite produced with its participation. The parameters included a high flexural strength (above 24.7 MPa) and a relatively low melting point (+80 °C), lower than that of polypropylene fibres (+120 °C).

The concrete was developed using Portland cement CEM I 42.5N CEMEX Cemex, Chełm, Poland. The manufacturer’s sheet specifies that the cement has stable chemical and physical attributes with a suitable setting time, high early and final strength parameters, a low alkali content, and a high resistance to aggressive chemical agents. Table 2 showcases the physical and chemical attributes of the cement.

The concrete additive used was Silica dust SILIMIC, Re Alloys, Łaziska Górne, Poland. The manufacturer’s description posits that SLIMIC comprises fine dust particles with a diameter 100 times smaller than the average grain size of cement. The replacement of 15% of cement with microsilica improved the impermeability of the concrete and was difficult to attain using other methods. The primary features of the microsilica, as documented in the technical sheet, are showcased in Table 3.

Concrete plasticizer FAST-MIX PRIMACOL PROFESSIONAL, (Unicell International, Wasilków, Poland) was used as a plasticizing admixture. Based on the manufacturer’s sheet, this admixture is a highly potent plasticizing plasticizer that distributes the cement particles in the concrete mix. The manufacturer specifies that the concrete plasticizer increases the frost resistance and water tightness of hardened concrete. The primary technical parameters of the admixture, based on the technical sheets of such plasticizers, are shown in Table 4.

The research composites were designed using an experimental method. An assumption held was that the starting point was to design a composite filled with the maximum agricultural mesh waste, designated as NET100%. First, a slurry was designed that would be liquid enough for the dosed mesh fibres to ensure that they were distributed evenly in the slurry. The dosage of the admixture and microsilica addition to the cement was influenced by the recommendations from the manufacturer. The analytical work was undertaken based on the author’s method, involving successive approximations. The weighed dry ingredients were mixed in a vessel where water portions were increased successively using the HEIDMANN GEKO 2300 (Geko, Radomsko, Poland) mixer. After stirring for about 1 min, a trial addition of recyclate was made without interruption of the mixing process. The cement slurry mixture was considered as optimum when the slurry surrounded all free fibres of the recyclate. The cement slurry was filled with the recyclate to the maximum degree until the slurry evenly surrounded all waste fibres. A 5 min length of the mixing cycle between the slurry and recyclate was also used. Table 5 showcases the composition of the slurry mix, where the quantities of the substrates used during slurry testing and their conversion to a working mix for a 1 m^3^ concrete mix are displayed.

The next step was to prepare a control mix (CONTR) containing traditional sand and gravel fillers. It was assumed that the proportions of 0–2 mm of sand and 2–4 mm of gravel used would be 1:2. The aggregate prepared in this way was to form the filler for the same slurry as that developed for the NET100% mix. The final composition of the control slurry, in relation to NET100%, and the full volume of the aggregate used at the time are presented in Table 6.

In the subsequent test series, the recycled agricultural net was utilised as an additive to modify the features of the control composite. The amount of additive increased from a value of 20 kg/m^3^ of the mixture for the NET20 series to 40 for the NET40 series, 60 for the NET60 series, and, finally, 80 for the NET80 series. This gradual additive increase was influenced by a desire to assess the impact of the amount of recyclates on the studied parameters of the composites. Table 7 showcases the compositions of the mixes, which include the control and the recyclate comprising the filler of the composite.

### 2.2. Testing Procedures

The samples in the study were prepared using a similar method, where the production process was similar to that used during the mix formulation. During the first phase, the cement slurry and net fibres were prepared in separate containers. Subsequent portions of waste material were incorporated into the slurry using the HEIDMANN GEKO 2300 (Geko, Radomsko, Poland) rotary mixer. The mixing cycle length of the slurry with the recyclate was approximately 5 min. The study considered 10 samples from each test batch during testing. The samples were subjected to moisture treatment by immersion in water, one day after munding. After 3 days, the samples were removed from the moulds and stored in a closed vessel under high humidity conditions. All prepared concrete mixtures were required to undergo an evaluation of consistency, referred to as the standard test based on the PN-EN 12350-2:2011 [130]. Volumetric density was tested on prismatic samples measuring 4 × 4 × 16 cm. The samples were measured using a meter and weight on a scale based on the EN 12390-7:2011 [131]. Water absorption was further tested on 4 × 4 × 16 cm samples. The water absorption was computed according to [132]. The method involved the ratio of the amount of water the composite absorbed to the dry composite, expressed as a percentage.

The flexural strength of the three-point scheme was tested according to the method proposed by [133]. Moreover, specimens measuring 4 × 4 × 16 cm were prepared for the test, and the compressive strength of the specimens was tested according to the method [134]. The 4 × 4 × 4 cm specimens were tested after the specimens were broken during the flexural strength test. The strength test was conducted on a MATEST 2000 (Matest S.p.A., Arcory, Italy) testing machine, with a 0–300 kN strain gauge attachment also from MATEST, model: C089PN468, factory number: C089PN468/AA/0001.

The thermal testing of the samples involved loading the specimens with temperatures simulating a fire phenomenon. The main element of the test stand for the influence of high temperatures on the prepared composites was a chamber furnace corresponding to those commonly used for firing ceramics. The cylinder-shaped furnace was heated by a gas burner that controlled the temperature inside. A thermocouple connected to an electronic temperature-measuring set was installed in the furnace. The samples were placed in the furnace in successive batches and loaded with the same temperature distribution, simulating real fire conditions, according to [135]. The furnace temperature rose rapidly from 20 °C to 1000 °C in 150 min (assumed 120 min), followed by an isothermal annealing process at 1000 °C lasting a further 60 min. After this time, the furnace burner was turned off and the samples remained in the furnace until they had cooled completely.

The final tests conducted were microscopic studies that involved a Scanning Electron Microscope and Energy-Dispersive X-ray Spectroscopy. The initial process considered the immersion of the resin and, thereafter, a slow-speed blade was utilized to cut through the entire prepared test material. As a result, this led to observing the internal structure of the composite. The TESCAN VEGA (Tescan Group, a.s., Kohoutovice, Czech Republic) Compact LMH equipment was also utilized to acquire micrographs. A secondary electron detector was also employed for images and an EDAX (Edax, Pleasanton, CA, USA) detector with an Si3N4 window was used for elemental analysis. Essence TM (Agilent, Santa Clara, CA, USA) suite was also used for the assessment of the images and elemental analysis.

## 3. Research Results and Analysis

In terms of the analysis of the consistency test results, it was demonstrated that the addition of the net worsened the workability of the concrete mixture. The assessed density of the mix showed a densely plastic consistency, and corresponded to a cone drop (S3) of 100 mm. However, the slurry used to prepare the composites revealed an increase in density with the addition of aggregate when producing the control mix (CONTR). This phenomenon was easily explained, because the sand and gravel aggregate was a saturated aggregate that absorbed water from the slurry. Although the recycling additive itself was not absorbent, its fibre form decreased the workability of the mix. Even a small addition of net (NET20) made the mix less workable, and this corresponded to a cone drop (S2) of 80 mm. Similarly, increasing the addition allowed this consistency (S2) to be maintained, but the cone drop decreased to a result of around 55 mm for the NET80 mix. When the net was used as filler in the NET100% test series, the consistency tested was also dense and corresponded to a cone drop of S2, close to that presented by the NET80 mix.

The results of this research work related to the results presented in the literature only confirmed the negative effect of plastic recyclates on the workability of concrete [87,88,89,90,91,92,93]. The presented results are similar to those presented by Skominas et al. [94], who, in their research work, proved that replacing 15% of fine aggregate with plastic resulted in a 40% lower workability. In the present study, the addition of 80 kg/m^3^ resulted in a 45% reduction in cone drop—from 100 mm for the CONTR mix to 55 mm for the NET80 mix.

**Figure 2:**
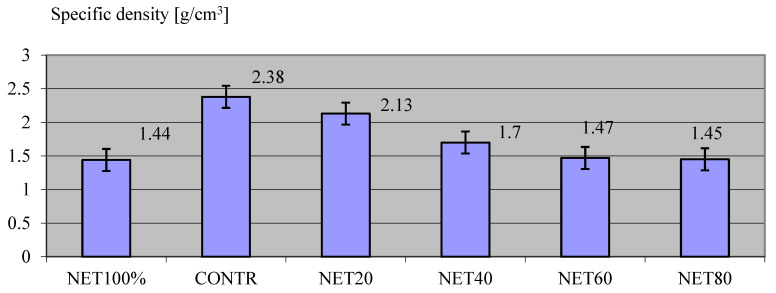
Results of specific density testing of composites.

Figure 2 shows the results of the specific density of the composites.

The assessed specific density of the composites was highest for the control sample (CONTR) without the recycled agricultural net content. In these samples, it was 2.38 g/cm^3^. A much lower value was presented by the NET20 composite samples with the addition of 20 kg/m^3^ of recyclate. The value for them was 2.13 g/cm^3^, and the difference in density between the control samples (CONTR) and NET20 was 10.50%. For the other samples, sequentially, the differences in values concerning the control sample (CONTR) were significantly higher, and the density values for the following series were reported as: for the NET40 series—1.70 g/cm^3^, for NET60—1.47 g/cm^3^, and the NET 80 series—1.45 g/cm^3^. For the test series, in which the entire composite was filled only with NET100% agricultural waste net, the volumetric density value was as much as 39.5% lower (1.45 g/cm^3^) than that for the CONTR control mix.

In the case of this technical parameter, the results of this research were similar to those presented in the literature [95,96,97,98,99], only confirming the observations of other authors and the principle of a decrease in the value of volumetric density with the addition of plastic recyclate. In assessing the degree of decrease in specific density, a reference can be made to the work of Ismail and Al-Hashmi [100] who, after testing samples containing 10%, 15%, and 20% plastic waste, showed that the density of the composite decreased by 5%, 7%, and 8.7%, respectively. In the case of our study, the addition of the NET20 series resulted in a density reduction of the aforementioned 10.5%, so the result was close to that presented by the cited authors.

**Figure 3:**
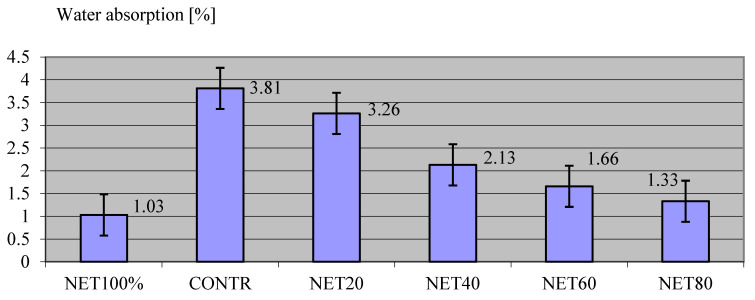
Results of the saturation test of the composites.

Figure 3 shows the results of the saturation test on the composites.

Testing the absorbability of the composites revealed that, when completely immersed in water, the control samples (CONTR) without recyclate absorbed more water, and the absorbability value for this series of samples was reported as 3.81%. The least absorbent samples were those in which net recyclate made up the total NET100% composite fill. The wettability value for this composite was 1.03%, which was 2.78% lower than that of the control composite (CONTR). For successive test series, in which the recyclate was only a dispersed reinforcement, the absorbability values decreased as the amount of recyclate increased. For subsequent test series, NET20 had a value of 3.26%, NET40 was 2.13%, NET60 was 1.66%, and NET80 was 1.33%. The results of this study were easy to predict. Both the cement stone and the aggregates are relatively highly absorbent materials compared to the non-absorbent recyclate. Thus, as the composite was filled with the waste net, part of its volume was filled with non-absorbent matter. Eventually, with the same volume of specimens in successive test series, the content of absorbent material in the specimens that made up the composite decreased, so the overall absorbency value for the composite also decreased. Relating this observation to the literature confirms the observations observed by other authors. Meena et al. [101], in their similar study, confirmed the observed rule. In addition, it has been revealed that lower water absorption will also have the effect of reducing the permeability of the composite, as well as increasing its durability [102,103], especially in winter conditions and exposure to cyclic freezing and thawing processes. Therefore, less absorbent concretes are more durable and are not subject to cracking due to freezing water increasing in volume inside them.

**Figure 4:**
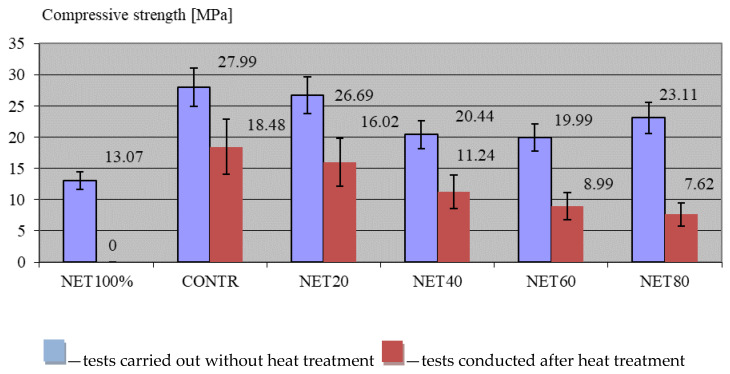
Compressive strength results of the composites.

Figure 4 presents the results of the compression strength tests on the composites. To make the values obtained easier to perceive, the graphs show both the results of testing the specimens under normal temperature conditions (blue bars) and the results of testing the composites after loading them with rapidly increasing temperatures (red bars).

The result of the compression strength test for standard temperatures was in line with the assumptions set out in the research. The fibres of the recycled net, similar in structure to textile fibres, which do not have a high compressive strength, per se, caused a decrease in the value of this parameter with increasing amounts of fibres in successive test series. The value assessed for the CONTR control composite was 27.99 MPa. The addition of recyclate (20 kg/m^3^) led to a 4.65% decrease in the NET 20 composite, and a final value of 26.69 MPa was recorded. In subsequent test series, the compressive strength value was lower than the control composite by 26.98% (20.44 MPa) for the NET40 composite, by 28.56% (19.99 MPa) for the NET60 composite, and slightly less by 17.45% (23.11 MPa) for the NET80 composite. As expected, the composite that had no aggregate and consisted of cement slurry and the NET100% recycled filler had an equally low compressive strength. Here, the value of this parameter was 13.07 MPa, which was 53.31% lower than that of the CONTR control composite.

The results of this study confirmed observations noted by other research teams, where it was found that the addition of plastics reduced the concrete’s compressive strength, tensile strength, modulus of elasticity, and unit weight [104,105]. Pereira et al. [106] similarly proved that the main influence on the compressive strength of a composite with recyclate was due to the volume that the recyclate occupied in the concrete mix, and as the volume of recyclate increased, the strength also decreased.

**Figure 5:**
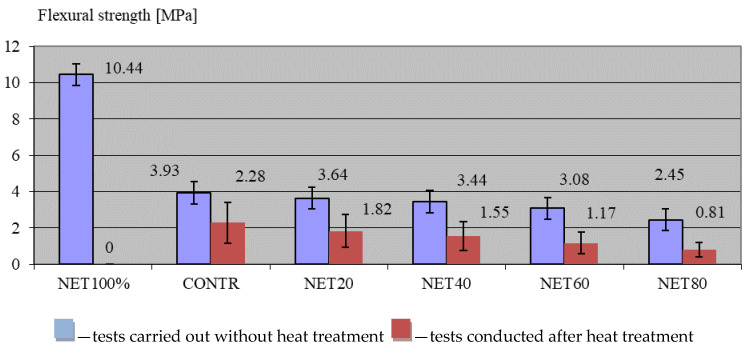
Flexural strength results of the composites.

Figure 5 presents the results of flexural strength tests on the composites.

**Figure 6:**
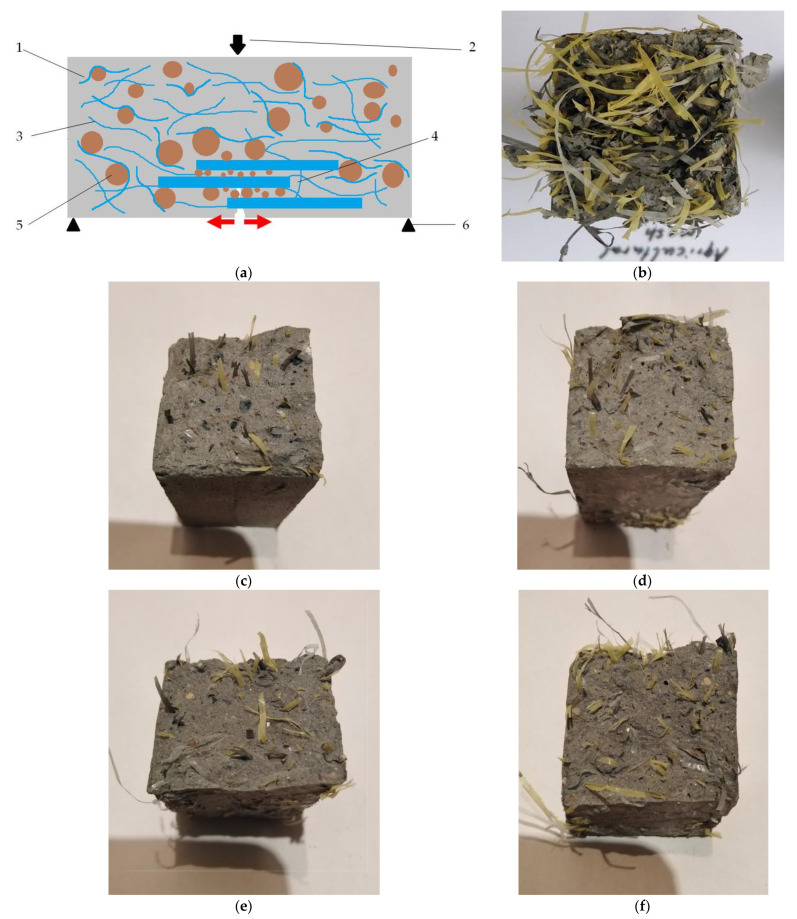
(**a**) Hypothetical phenomenon of recyclate fibres “hanging” on aggregate grains. Designations: (1)—cement stone, (2)—bending force, (3)—recycled reinforcing fibres, (4)—fibre sliding over aggregate scheme, (5)—aggregate grains, (6)—supports in (**a**) three-point bending scheme. (**b**)—image of NET100% composite after flexural testing, (**c**)—image of NET20 composite, (**d**)—image of NET40 composite, (**e**)—image of NET60 composite, and (**f**)—image of NET80 composite.

The results of the flexural strength test under standard conditions were quite non-obvious. The reference point here was a control composite (CONTR), for which the value of this parameter was 3.93 MPa. Although it was assumed that the fibres of the high-tensile agricultural net (24.7 MPa) would be the dispersed reinforcement, which would increase the flexural strength of the composite with increasing amounts of recyclate, the value of this parameter decreased. Thus, in successive test series, the flexural strength value was lower than the control by 7.38% (3.64 MPa) for the NET20 composite, 12.47% (3.44 MPa) for the NET 40 composite, 21.63% (3.08 MPa) for the NET60 composite, and 37.66% (2.45 MPa) for the NET80 composite. A surprising result was also obtained for the composite filled with recycled agricultural net. The flexural strength value was very high at 10.44 MPa. In comparison, the value obtained for the CONTR control composite was 62.36% lower. Analysing the results described, it was concluded that this property of the composite could potentially have been negatively affected by the combination of aggregate and recyclate. An explanation for this occurrence was that the fibres of the recyclate may have been in direct contact with the aggregate in the mix. There may have been no cement slurry between these phases, and zones without binding forces may have formed. Figure 6a showcases the weakening of the composite by the recyclate. The aggregate fibres come into contact with the composite and slide without maintaining adhesion with the slurry. By filling the slurry itself with fibres, a lightweight composite with very high flexural strength parameters was eventually obtained, as shown in Figure 6b. The fibres here, by not ‘hanging’ on the aggregate, fulfilled their hypothetical task of reinforcement and increased the value of this parameter.

The comparison of the results of this study to the past literature reveals that the flexural strength characteristics depend mainly on the type of recyclate. Despite the frequently observed decrease in strength, the literature studies indicate that the addition of recyclate can have a positive effect on the flexural strength and that it can increase with an increase in the proportion of plastic [107,108,109,110,111]. However, these observations mainly concern plastics made from higher-strength materials than HDPE.

The results of the study on the resistance of the composites to rapidly increasing temperatures were similar to those reported in the literature for analyses carried out with polypropylene fibres [80,81]. No spallation occurred on any of the samples tested during annealing. This could probably also have been due to the low strength value of the composites tested and the control composite. The phenomenon of thermal spalling occurs most frequently in compact and tight composites of a high strength [73,74,75,76,77]. In such cases, water confined in the capillaries by boiling causes the explosive rupture of the capillaries and rapid detachment of the composite particles. In the case of low-strength concretes and relatively high porosity, the reference of which is the tested absorbability, this phenomenon does not increase. Despite this, an organoleptic examination of the fracture of annealed specimens has already shown that, during annealing, waste fibres were destroyed and hollow channels formed inside the specimens, which, in airtight and compact composites, could provide a route for water vapour migration.

The results of the strength tests carried out on specimens subjected to thermal loading, as showcased in Figure 4 and Figure 5, revealed a negative effect of the fibres on the composites modified with them. All composites showed a significant decrease in both compressive and flexural strength after annealing. For the control composite, the decrease in compressive strength was 34% (27.99 MPa before and 18.48 MPa after annealing), and in flexural strength, was 42% (3.93 MPa before and 2.28 MPa after annealing). The addition of waste net fibres increased the differences for the annealed specimens. Successively, for the NET40 series, these differences were 50 and 40%, for the NET40 series they were 55 and 45%, for the NET60 series they were 62 and 55%, and finally, for the NET80 composite, these differences were a 67% decrease in compressive strength and a 67% decrease in flexural strength. Interestingly, the composite filled with NET100% could not remain tested because the specimens were destroyed during the test. This was easily predictable, because the destruction of a large volume of the component resulted in a very large number of air voids, and the remaining small amounts of grout were crushed.

A comparison of the findings to the literature also confirmed the observations made by other authors. In general, the addition of plastic fibres (like polypropylene fibres [80,81]) protects the composite against spallation. However, composites with hollow fibres inside them always show a decrease in strength parameters after annealing. The effect of their use is expected to be the non-occurrence of cracks and damage, as demonstrated, for example, by Mohammadhosseini and Yatim [112]. Analysing the results of their work, they found that the addition of plastic fibres had a positive effect on composites exposed to 800 °C. The additive was effective in reducing thermal spalling. Similar conclusions were drawn from the study by Girardi et al. [113]. In this case, composites containing 0.5% recycled fibres after exposure to temperatures of the order of 450 °C had no damage, while the same concrete without fibre additives cracked at temperatures of the order of 150 °C. Furthermore, in [114], the authors proved that composites containing plastics have a lower thermal conductivity, and in [115], the effect of the melting of synthetic fibres was confirmed, which was also observed in the present case.

The last of the tests carried out were microscopic studies. The samples of the NET80 composite containing sand–gravel aggregate after preheating at temperatures below 200 °C and the NET100% composite samples after annealing and complete fibre melting were tested.

**Figure 7:**
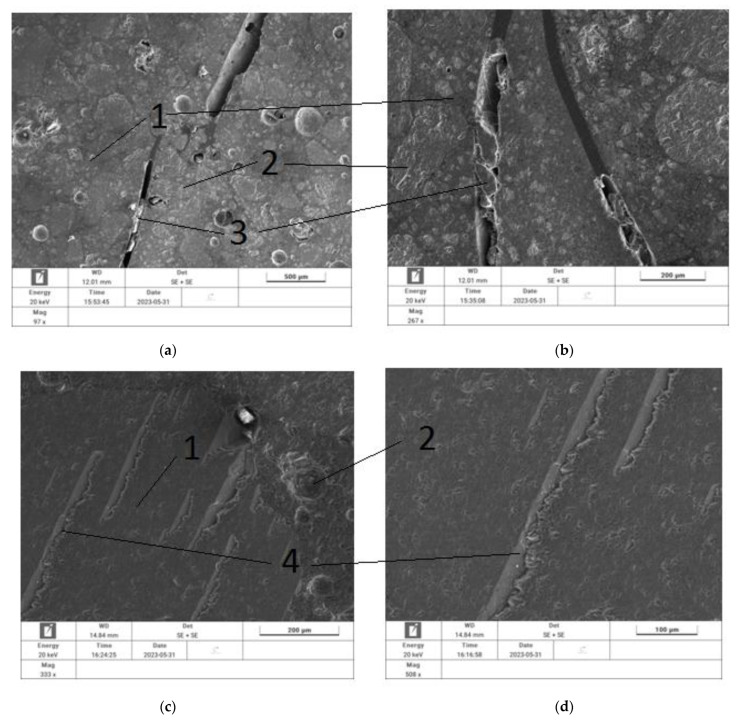
SEM images of a cementitious composite containing recycled agricultural net fibres: (**a**,**b**)—fibres images of NET80 composite containing sand–gravel aggregate after initial annealing at temperatures below 200 °C, (**c**,**d**)—images of NET100% composite after annealing and complete melting of the fibres. (1)—cement slurry, (2)—aggregate, (3)—recyclate fibres, (4)—voids caused by melting of the recyclate.

Figure 7 presents the images obtained during the analyses.

Figure 7a,b show both aggregate, cement stone, embedded fibres, and air voids in the cement stone after the fibres of the waste composite net melted. The images presented show that, as expected, the rather large filling of the composite containing aggregate with the waste net may have led to grains of fine aggregate adhering directly to the net, which reduced the bond between the fibres and the cement stone. Partially burnt fibres can also be seen due to elevated temperatures. In Figure 7c,d, only the air voids in the slurry caused by the scorching of the net fibres are already visible.

**Figure 8:**
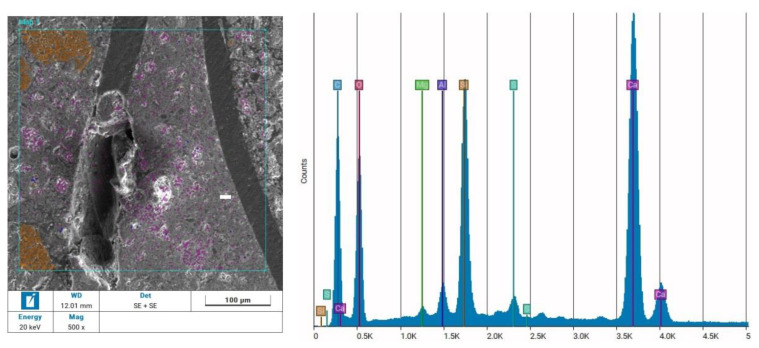
Results of an elemental composition study of the NET80 composite performed using energy-dispersive X-ray Spectroscopy.

To confirm the conclusions read from the microscopic images, an analysis of the elemental composition of the zones highlighted in the photographs was also carried out. The results of this study are presented in Figure 8.

An analysis of the elemental composition of the zones both analysed as cement stone and the zones analysed as recyclate fibres and aggregate confirmed the validity of the interpretation of the earlier photographs. The main component of the aggregate was silicon Si, marked in brown in the photograph, which was interpreted as the building block of the sand grains used to prepare the composite. The main component of the cement stone was calcium Ca (colour purple), which is the main component of cement slurries and limestone aggregates. An analysis of the image section, considered as a fibre, proved that this part of the test sample was mainly composed of carbon (C), marked in blue. This was also in line with conjecture, as the main constituent of high-density polyethylene HDPE is this chemical element—(C_2_H_4_)_n_.

## 4. Conclusions

The advancement of agricultural net technologies has allowed more farmers to embrace them. However, with increased use of these nets, more waste is generated, for instance, in cereal crop farming. To address the high levels of waste produced from agricultural nets, the current research examined the unexplored possibilities of recycling bale-wrapping mesh in its secondary use as an additive and filler for cement concrete. The examination of different studies proved the hypothesis in the study, where cleaning and grinding agricultural nets generated fillers and dispersed reinforcement within the cementitious composites.

The results of the test work demonstrated that the addition of a net worsened the workability of the concrete mixture, and with increasing amounts of additive, the consistency of the mixture could change from liquid to dense plastic. The assessed specific density of the composites was highest for the control sample (CONTR)—with no recycled agricultural net content—and this decreased as the amount of recycled net increased. For the test series, in which the entire composite was filled only with NET100% recycled agricultural net, the value of the bulk density was lower by as much as 39.5% (1.45 g/cm^3^) than that for the CONTR control mixture (2.38 g/cm^3^). Testing the absorbability of the composites demonstrated that the absorbability of the samples decreased as the amount of recyclate increased. The least absorbent samples were those in which the net recyclate made up the total NET100% composite fill. The absorbability value for this composite was 1.03%, which was 2.78% lower than that of the CONTR control composite (3.81%). The flexural strength of the composite decreased with increasing amounts of recyclate. In subsequent test series, the flexural strength value was lower than the control CONTR (3.93 MPa) by 7.38% (3.64 MPa) for the NET20 composite, by 12.47% (3.44 MPa) for the NET 40 composite, by 21.63% (3.08 MPa) for the NET60 composite, and by 37.66% (2.45 MPa) for the NET80 composite. The flexural strength value of the 100% net-filled composite (NET100%) was very high at 10.44 MPa, whereas the value obtained for the CONTR control composite was 62.36% lower. The results of the compressive strength test showed a decrease in this parameter with increasing amounts of additive. The value assessed for the CONTR control composite was 27.99 MPa. The addition of recyclate in an amount as low as 20 kg/m^3^ of the mix resulted in a 4.65% decrease in the value of this parameter (26.69 MPa). In subsequent test series, the compressive strength value was lower than that of the control composite by 26.98% (20.44 MPa) for the NET40 composite, by 28.56% (19.99 MPa) for the NET60 composite, and by 17.45% (23.11 MPa) for the NET80 composite. As expected, the composite that had no aggregate and consisted of cement slurry and NET100% recycled filler had an equally low compressive strength. Subsequently, the value of this parameter was 13.07 MPa and was as much as 53.31% lower than that of the control composite. The results of the study on the resistance of the composites to rapidly increasing temperatures were similar to those reported in the literature for analyses carried out with polypropylene fibres. All composites showed a significant decrease in both compressive and flexural strength after annealing. For composites with 80% recycled net added, these differences showed a 67% decrease in the strength parameters between those obtained before and after annealing. The NET100% samples filled with the recycled net were destroyed during this test. SEM imaging proved that the fibres from the shredded NET formed a dispersed reinforcement in the NET100% composite, bonding tightly to the cement stone, and that there were no anomalies in the bonding zone that deteriorated the contact zone. The disappearance of fibres at high temperatures was also confirmed by microscopic studies.

The results of the analyses carried out conclusively proved that the best use of waste net fibres is as a filler in lightweight cementitious composites. NET100% composites are characterized by a low self-weight, low absorption, high flexural strength, and satisfactory compressive strength. This type of proposed solution can be justified both ecologically and economically. The environmental advantages of this type of solution are that there is no need for detailed waste treatment or thermal treatment, which releases detergents and heat deposits into the environment. These processes are also cheaper for entrepreneurs, as they can be carried out locally at concrete production plants, and the adaptation of the waste does not require any special techniques, apart from grinding the recyclate.

## Figures and Tables

**Table 1:** Technical parameters of HDPE net taken from the material manufacturers’ resources.

Feature/Evaluation Method	Value
Chemical composition/PN-C-89280:1986 [116]	(C_2_ H_4_)_n_
Volumetric density/PN-EN ISO 1183-2:2006 [117]	0.941–0.965 g/cm^3^
Thermal conductivity/PN-EN ISO 1872-2:2008 [118]	0.46–0.51 W/m- K
Tensile strength/PN-C-04243:197 [119]	24.7 MPa
Relative elongation at yield stress/PN-EN ISO 1872-2:2008 [118]	>9.1%
Temperature resistance/PN-C-89280:1986 [120]	Above −50/below +80

**Table 2:** Physical and chemical parameters of the cement from the manufacturer’s product sheet.

Feature/Evaluation Method	Unit	Average Score	Requirements
Initial setting time/EN 196-3:2009 [121]	min	233	>60
Final setting time/EN 196-3:2009 [121]	min	291	
Water efficiency/EN 197-1:2012 [122]	%	27.5	
Constant volume/EN 197-1:2012 [122]	mm	1.1	<10
Specific surface area/EN 196-6:2011 [123]	cm/g^2^	3688	
Compressive strength: after 2 days/EN 197-1:2012 [122]	MPa	23.9	<10
Compressive strength: after 28 days/EN 197-1:2012 [122]	MPa	55.9	42.5–62.5
Chemical analysis: SO_3_/EN 196-2:2006 [124]	%	2.77	<3.0
Chemical analysis: Cl/EN 196-2:2006 [124]	%	0.070	<0.10
Chemical analysis: Na_2_Oeq./EN 196-2:2006 [124]	%	0.53	<0.6

**Table 3:** Basic properties of microsilica based on the product datasheet.

Parameter	Unit	Value	Evaluation Method
Form	-	fine powder	Visual
Colour	-	grey	Visual
Fragrance	-	odourless	-
Density	g/cm^3^	2.05	PN-EN 1097-6:2013-11 [125]
Bulk density	g/cm^3^	1.1	PN-EN 1097-3:2000 [126]
Alkalinity	pH	less than 11.5	PN-EN ISO 10523:2012 [127]

**Table 4:** Basic properties of admixture based on the manufacturer’s technical sheet.

Property/Evaluation Method	Description
Form	Liquid
Chloride content/PN-EN 196-2:2006 [124]	<0.1%
Alkali content/PN-EN ISO 10523:2012 [127]	<2.0%
Compressive strength/PN-EN 197-1:2012 [122]	After 7 days, concrete tested ≥110% control concrete After 28 days, concrete tested ≥110% control concrete
Air content/PN-EN 934-2+A1:2012 [128]	Test mixture ≤ 2% by volume above the content in the mixture control
Water reduction earnings/PN-EN 934-2+A1:2012 [128]	In the test mixture ≥5% w compared to the control mixture
Density (20 °C):/PN-EN 1097-6:2013-11 [125]	1.075 ± 0.02 kg/dm^3^
pH:/PN-EN ISO 10523:2012 [127]	5 ± 1
Grain size/PN-EN 933-1:2000 [129]	0.15 µm

**Table 5:** Quantities of substrates used during the test work and their conversion into a 1 m^3^ concrete working mix.

	Quantity of Substrate in kg Used for the Test Batch	Density Theoretical Substrate in kg/m^3^	Volume in m^3^ of Trial Work	Conversion Factor per kg/m^3^ of Mixture	Mix Amount of Substrate in kg/m^3^ of Mixture	Density of the Substrate in kg/m^3^	Component Volume in m^3^
Cement CEM I 42.5R CEMEX	1.340	3100.00	0.000432	412.69	553.00	3100.00	0.1784
Agricultural net fibres	1.270	950.00	0.001337	412.69	524.12	950.00	0.5517
Water	0.595	1000.00	0.000595	412.69	245.55	1000.00	0.2456
FAST-MIX	0.030	1050.00	0.000029	412.69	12.38	1050.00	0.0118
Microsilica	0.067	2200.00	0.000030	412.69	27.65	2200.00	0.0126
		Total	0.002423		136.70		1.0000

**Table 6:** Composition of control mix containing conventional sand and gravel aggregate.

Component	Quantity of Substrate in kg	Density of the Substrate in kg/m^3^	Volume in m^3^
Cement CEM I 42.5R CEMEX	553.00	3100.00	0.178387
Sand 0–2 mm	48.00	2480.00	0.193548
Gravel 2–4 mm	960.00	2650.00	0.362264
Water	245.55	1000.00	0.245550
FAST-MIX	12.38	1050.00	0.011790
Microsilica	27.65	2200.00	0.012568
Total	2278.58		1.004108

**Table 7:** Compositions of all mixtures developed during the analyses.

Component/Quantity in kg/m^3^	NET100%	CONTR	NET20	NET40	NET60	NET80
Cement CEM I 42.5R CEMEX	553.000	553.000	553.000	553.000	553.000	553.000
Sand 0–2 mm	-	480.00	480.00	480.00	480.00	480.00
Gravel 2–4 mm	-	960.00	960.00	960.00	960.00	960.00
Water	245.55	245.55	245.55	245.55	245.55	245.55
FAST-MIX	12.38	12.38	12.38	12.38	12.38	12.38
Microsilica	60.00	60.00	60.00	60.00	60.00	60.00
Agricultural net fibres	524.12	-	20.00	40.00	60.00	80.00

## Data Availability

The original contribution presented in the study are included in the article, further inquiries can be directed to the corresponding author.
